# Effects of Harmful Algal Blooms on Fish and Shellfish Species: A Case Study of New Zealand in a Changing Environment

**DOI:** 10.3390/toxins14050341

**Published:** 2022-05-14

**Authors:** Anne Rolton, Lesley Rhodes, Kate S. Hutson, Laura Biessy, Tony Bui, Lincoln MacKenzie, Jane E. Symonds, Kirsty F. Smith

**Affiliations:** 1 Cawthron Institute, 98 Halifax Street East, Nelson 7010, New Zealand; lesley.rhodes@cawthron.org.nz (L.R.); kate.hutson@cawthron.org.nz (K.S.H.); laura.biessy@cawthron.org.nz (L.B.); tony.bui@cawthron.org.nz (T.B.); lincoln.mackenzie@cawthron.org.nz (L.M.); jane.symonds@cawthron.org.nz (J.E.S.); kirsty.smith@cawthron.org.nz (K.F.S.); 2 Centre for Sustainable Tropical Fisheries and Aquaculture, College of Science and Engineering, James Cook University, Townsville 4811, Australia

**Keywords:** green-lipped mussels, oysters, finfish, salmon, aquaculture, climate change

## Abstract

Harmful algal blooms (HABs) have wide-ranging environmental impacts, including on aquatic species of social and commercial importance. In New Zealand (NZ), strategic growth of the aquaculture industry could be adversely affected by the occurrence of HABs. This review examines HAB species which are known to bloom both globally and in NZ and their effects on commercially important shellfish and fish species. Blooms of *Karenia* spp. have frequently been associated with mortalities of both fish and shellfish in NZ and the sub-lethal effects of other genera, notably *Alexandrium* spp., on shellfish (which includes paralysis, a lack of byssus production, and reduced growth) are also of concern. Climate change and anthropogenic impacts may alter HAB population structure and dynamics, as well as the physiological responses of fish and shellfish, potentially further compromising aquatic species. Those HAB species which have been detected in NZ and have the potential to bloom and harm marine life in the future are also discussed. The use of environmental DNA (eDNA) and relevant bioassays are practical tools which enable early detection of novel, problem HAB species and rapid toxin/HAB screening, and new data from HAB monitoring of aquaculture production sites using eDNA are presented. As aquaculture grows to supply a sizable proportion of the world’s protein, the effects of HABs in reducing productivity is of increasing significance. Research into the multiple stressor effects of climate change and HABs on cultured species and using local, recent, HAB strains is needed to accurately assess effects and inform stock management strategies.

## 1. Introduction

Harmful algal blooms (HABs) can cause significant impacts on human and animal health due to the production of toxic or bioactive compounds, mucilage, aggravating cellular structures like spines, or oxygen depletion of seawater [1,2,3,4]. Occurring when favourable environmental conditions exist for abundant algal growth (reviewed in [5]), HABs can have major economic and environmental repercussions [6,7,8,9]. For example, in the Gulf of Mexico, USA, annual blooms of the nonthecate dinoflagellate *Karenia brevis* (=“red tide” alga), have caused massive problems for recreational fisheries, tourism, the aquaculture sector, and the wider environment through the production of potent marine neurotoxins known as brevetoxins, BTXs [10]. The major impact of *K. brevis* blooms on the aquaculture industry has been from closure of harvest areas containing brevetoxic shellfish and toxic effects of *K. brevis* exposure also directly impact commercially fished bivalve species [11,12,13,14].

The direct effects of HABs on cultured fish and shellfish species can be substantial and are likely to become more significant considering increasing climate anomalies and as the farmed supply of seafood grows [15]. The early life stages of cultured species are particularly susceptible to harmful algae and their associated toxic compounds [12,16,17,18,19,20,21,22,23]. Most farmed species are confined and cannot escape blooms. This makes them extremely vulnerable to HABs, which are often sporadic and severe, and can cause losses of entire cohorts [24,25,26]. For example, in 2019, a bloom dominated by the ichthyotoxic haptophyte, *Chrysochromulina leadbeateri*, occurred in the Nordic fjords and was accompanied by the death of around seven and a half million farmed Atlantic salmon, *Salmo salar*, valued at over US$90M [27]. *Chrysochromulina leadbeateri* has been reported in Aotearoa/New Zealand (NZ)’s temperate coastal waters; however, it has not been associated with fish mortality to date [28].

In addition to acute lethal effects, HABs can cause a suite of behavioural and physiological effects on shellfish and fish, affecting the whole organism, or impacting at the tissue, cellular, or molecular level e.g., [13,29,30,31,32,33,34,35]. These sublethal effects (reviewed in [2,3] and discussed in Section 2) are often overlooked but can result in decreased feeding and thus slower growth rates and increased susceptibility to disease, further reducing commercial productivity.

### New Zealand Aquaculture and HABs

In NZ, the aquaculture industry is worth nearly NZ$650M (=US$0.5B, [36]) and comprises three predominant species: the green-lipped mussel (Greenshell™ mussel/GSM, *Perna canaliculus*), king salmon (=Chinook, *Oncorhynchus tschawytscha*), and the Pacific oyster (*Crassostrea gigas*). In 2019, total annual aquaculture production in NZ was approximately 115,000 tonnes, 88% of which came from bivalve production (mainly GSM, 98,447 tonnes and Pacific oysters, 1871 tonnes) and 12% from king salmon production (nearly 15,000 tonnes; [37]). The government’s 2019 Aquaculture Strategy targets growth of the industry to NZ$3B (>US$2B) by 2030 [36]; however, a risk to this ambitious target is the occurrence of HABs and the uncertainty around the effects of HAB species and their associated toxins on cultured species.

New Zealand Marine Biotoxin Monitoring Programmes encompassing both commercial and non-commercial shellfish harvesting, have been operating since 1993, funded by industry and the NZ Government’s Ministry for Primary Industries. Samples are analysed for potentially toxic phytoplankton and/or biotoxins and these data are used to inform risk assessments on which seafood managers can base harvesting decisions. Seawater samples are routinely collected from approximately 100 specific sites throughout NZ for laboratory identification and enumeration of toxic micro-algae. A risk assessment is supplied to biotoxin regulators and farm managers within 24 h of sample receipt. Many of these sites are near shellfish and finfish farms to provide an early warning of HABs that may affect stocks. Some finfish farmers also carry out independent, routine phytoplankton monitoring on their leased sites. Despite the extensive monitoring programmes in place in NZ, there remain unknowns regarding which microalgal species or combinations of microalgae are harmful to aquatic animals and what their effects are.

**Figure 1:**
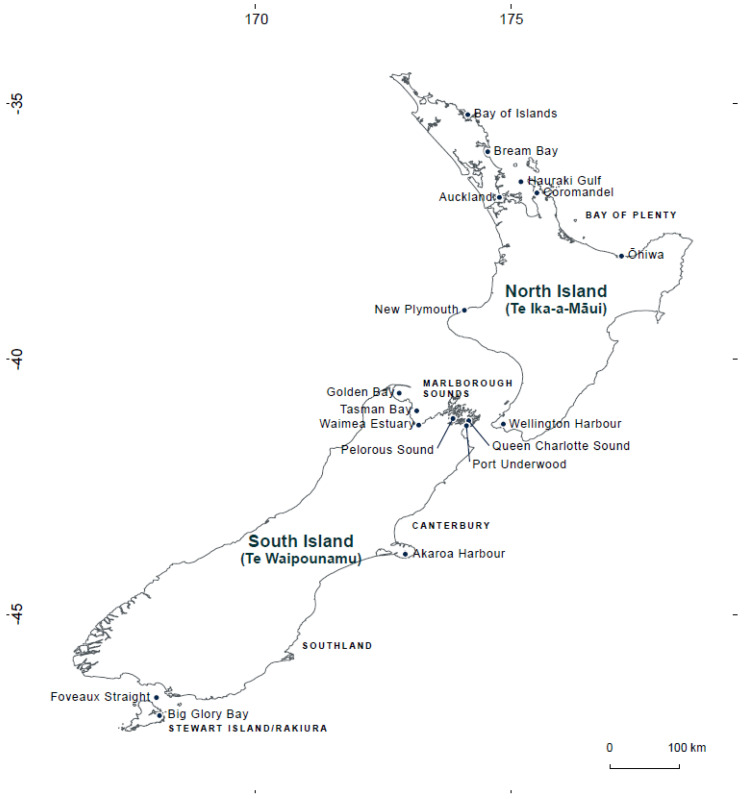
Map showing New Zealand locations mentioned in the manuscript.

Recent salmon mortality, severe declines in GSM spat settlement, and unexplained summer mortalities of GSM have been reported in NZ [38,39,40]. Although the causes of these events are not clear, HABs may be a contributing factor. Previous HAB events in NZ have impacted cultured species, e.g., [41,42,43,44] and HABs frequently occur in important growing areas for Pacific oyster, GSM, and king salmon (i.e., Southland, Stewart Island, Marlborough Sounds, Bay of Islands, Auckland, and Coromandel, Figure 1). Such events appear to be increasing in geographic range, duration, and frequency. In the Marlborough Sounds, novel HABs of the thecate dinoflagellate *Alexandrium pacificum* first appeared in Queen Charlotte Sound in 2011 [45], around the same time as a reduction in GSM spat-fall in traditional spat catching areas was first observed [38,40]. Blooms have since been detected in the neighbouring Pelorus Sound, Tasman Bay, and Golden Bay and are moving towards the West Coast of the South Island [25,45,46], Figure 1.

Common HAB species present in NZ and their geographic distributions have recently been reviewed in [25] and a comprehensive review on the risk of paralytic shellfish poisoning (PSP) toxins on shellfish aquaculture in NZ was carried out by [45]. To complement these works, the aim of this study was to identify the effects of HAB species which have bloomed in NZ and impacted on commercially important fish and shellfish species of relevance to the aquaculture industry. We also consider HAB issues that may be encountered in a changing environment. New Zealand locations mentioned in the text are shown in Figure 1.

## 2. Effects of Bloom Forming HAB Species on Species of Relevance to the NZ Aquaculture Industry

**Figure 2:**
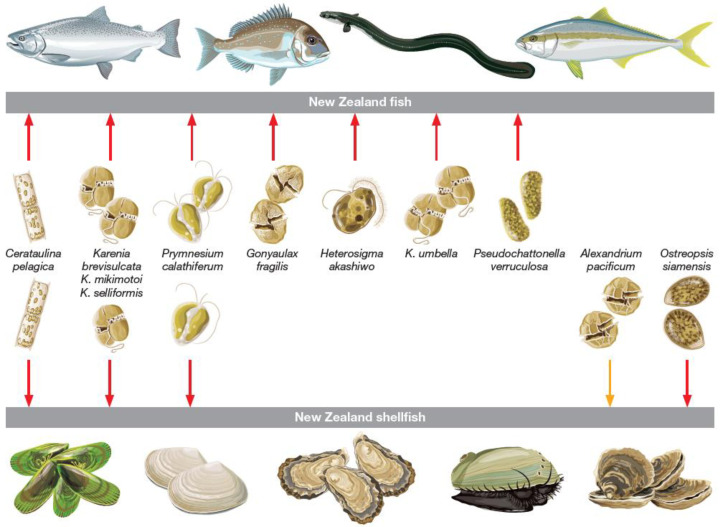
Known lethal (red arrows) and sublethal (orange arrow) effects of harmful algal species that have bloomed on fish and shellfish in New Zealand. Arrows indicate blooms that have impacted fish (e.g., *Heterosigma akashiwo*), shellfish (e.g., *Alexandrium pacificum*), or affected both fish and shellfish (e.g., *Karenia brevisulcata*). Image: Eden Cartwright, Bird Circus.com.

Several HAB-forming species present in NZ have been implicated in lethal or sublethal effects on commercially important fish and shellfish species (Figure 2, Table 1). Here, we consider these and other key genera including *Alexandrium*, *Gymnodinium*, *Karenia*, *Dinophysis*, *Pseudo-nitzschia*, and *Heterosigma*, *Fibrocapsa*, which have bloomed in NZ waters, and their impact on species of relevance to the aquaculture industry in NZ.

### 2.1. Alexandrium and Gymnodinium spp.

Blooms of *Alexandrium pacificum*, *A. minutum*, and *Gymnodiniun catenatum*, all thecate dinoflagellates, have been recorded in NZ coastal waters [25,45]. Cells of these species can produce paralytic shellfish toxins (PSTs), including saxitoxin and its many analogues, and other bioactive extracellular compounds (BECs) which can cause ichthyotoxic and negative effects on shellfish [16,66,67,132,133]. The presence of these other BECs, in addition to PSTs, can make it difficult to determine the causative compound of any cytotoxic effects.

Negative behavioural and physiological effects following exposure of adult shellfish species to ***A. pacificum*** have been reported by several authors [34,60,61,62,63,134], as have lethal and sublethal effects on the early life stages of scallops, *Argopecten irradians*, where toxicity was attributed primarily to the presence of unknown bioactive toxins rather than PSTs [64,65]. Within NZ, investigations into the effects of *A. pacificum* on cultured shellfish species have largely focussed on later life stages (Figure 2). Behavioural and physiological effects include erratic siphon activity of surf clams, *Paphies donacina*, and alterations in the clearance rate of bivalves, including scallops, *Pecten novaezelandiae*, and flat oysters, *Ostrea chilensis*, exposed to bloom concentrations of *A. pacificum* see [55,56,58,59]. Juvenile oysters, *O. chilensis* (~20 mm), were also paralysed, but still alive, during the first *A. pacificum* bloom that occurred in Queen Charlotte Sound in 2011 and lasted for 2 months [135]. Most of these animals eventually died, as the mantle over-grew the shell margins, and they could not close their valves after the bloom subsided. A reduction in adult byssus production [55], and spat (~2.5 mm) byssal pad formation and growth [57], have been reported in GSM exposed to *A. pacificum*, which has implications for the survival, attachment, and retention of wild and farmed spat. It remains unclear if negative effects are caused by ingestion of toxic cells (intracellular toxins), contact with extracellular toxins (PSTs or other), or cell surface contact.

During *A. pacificum* blooms in NZ, cells have been observed within salmon farms, but cell numbers have generally been low and no adverse effects on the health of the fish have been reported [45]. The neurotoxicity of PSTs, however, is well documented in fish [2]. They cause the impairment of sensor-motor function, such as loss of orientation, paralysis, and abnormal swimming behaviour, that affects both adult and larval fish survival [19] (and references there in).

Blooms of ***Alexandrium minutum*** have been investigated since 1993 in NZ, when the first incident of PSP contamination in shellfish was identified [136,137,138]. Since then, this dinoflagellate has been implicated in several incidents in NZ, which have required the closure of commercial shellfish harvesting in the Marlborough Sounds and human illness from recreational harvest in the Bay of Plenty [25,45,139]. *Alexandrium minutum* has not been associated with fish mortalities and investigations into the effects of *A. minutum* on shellfish in NZ are lacking.

There has been extensive study of the effects of *Alexandrium minutum* on Pacific oysters, *C. gigas*. Inflammatory responses are typically seen in the digestive gland and hemolymph of adult *C. gigas* exposed to bloom concentrations of *A. minutum* [68,69], and behavioural modifications can include reduced clearance and filtration rate, valve activity and increased shell micro-closures, genotoxic effects, and an increase in antioxidant and detoxification gene expression in the gills [70,71,72,73]. *Crassostrea gigas* exposed to toxic *A. minutum* during gamete ripening, showed negative impacts on sperm motility, larval size, and settlement of larvae produced from exposed parents [67,68]. Recent studies have provided a better understanding of the toxicity of *A. minutum* to bivalves [66,75], with BECs found to be responsible for the negative effects on the gills of adult *C. gigas*, whereas PSTs impact the digestive gland [66]. Direct exposure of the early life stages of *C. gigas* also negatively affected gamete quality, fertilization success, and subsequent larval development [16,17,67,74], with negative effects attributed primarily to BECs rather than PSTs.

In the Port River in South Australia, an *A. minutum* bloom in the late 1980s showed an ichthyotoxic potential for this dinoflagellate species [140]. It was evident that the ichthyotoxicity was not caused by PSTs and that the toxin(s) were most likely effective exocellularly. This has been suggested to be a defence or allelopathic mechanism [25]. While saxitoxin-like and brevetoxin-like activity was indicated by a neuroblastoma tissue culture assay, no BTXs were detected, and the toxic principle remains unknown [140].

One of the most harmful blooms to be documented in NZ was that of PST producing ***Gymnodinium catenatum*** [45]. Blooms started in the North Island in May 2000 and lasted until 2003, during which time they caused wide-spread contamination and closure of shellfish harvest areas [141]. There have been further blooms since that time [45].

Exposure of trochophore *C. gigas* to 10^7^ cells L^−1^ of *G. catenatum* for 10 h had no effect on larval survival [85], however; Pacific oyster spat (3 mm) exposed to a lower concentration of *G. catenatum* (3 × 10^5^ cells L^−1^) for 24 h, showed behavioural and physiological changes, such as reduced clearance rates, increased valve shell closures, and increased pseudofaeces production [83]. Following a longer, 14 d exposure of *C. gigas* spat, exfoliation and epithelial rupture and inflammation were recorded in the digestive gland and gill, as were increased expression of genes involved in antioxidant defence, cell detoxification, intermediate immune response activation, and stress responses [83,84]. Additional physiological responses, including paralysis and apoptosis of hemocytes, have also been recorded in scallops, *Argopecten ventricosus*, and *Nodipecten subnodosus* [86,87,88]. The effects of NZ *G. catenatum* strains on aquaculture species are yet to be determined.

Blooms of *Gymnodinium catenatum* have rarely been associated with wild fish kills worldwide (see [81,82]) and never in NZ. Although blooms of this species have been extensive in the North Island of NZ, they occur relatively infrequently and to date have not affected the main finfish aquaculture areas in the south [45].

### 2.2. Karenia spp.

Several species in the non-thecate dinoflagellate genus *Karenia* have been identified in NZ waters, including *Karenia papilionacea*, *K. selliformis*, *K. bidigitata* (=*K. bicuneiformis*), *K. mikimotoi*, *K. umbella*, and *K. brevisulcata*, of which some produce toxins such as BTX, BTX-like compounds, gymnodimine, and haemolytic glycolipids [142]. To date, *K. mikimotoi*, *K. selliformis*, *K. brevisulcata* and, more recently, *K. umbella* have formed and dominated blooms, with the former three species also implicated in large scale finfish and shellfish mortalities [90,96,97,98,110,142,143], Figure 2.

***Karenia mikimotoi*** is one of the most common HAB species in NZ waters and is regularly associated with losses of farmed fish, and commercial and wild shellfish worldwide (reviewed by [23,93,94]). Japanese pearl oysters, *Pinctada fucata martensii*, for example, have been severely affected by recurrent blooms (e.g., [144,145]). Unsurprisingly, the early life stages of this and other commercially important shellfish species have been shown to be susceptible, with toxicity speculated to be from unknown bioactive compounds [85,99,105,106,107,108,109]. Cells of *K. mikimotoi* have been shown to produce haemolysin, which elicits toxic effects upon direct contact with the cells, and the reduced clearance rates of these cells by commercially important species such as adult *C. gigas* and *M. galloprovincialis* may be for this reason [61,100,101,102]. Exposure to this dinoflagellate also affects the immune functions of shellfish [33,103,104].

A *K. mikimotoi* dominated bloom that occurred in 1992 to 1993 in the Hauraki Gulf was a turning point for the NZ seafood industry and resulted in the development of the monitoring programmes that exist today [146]. At the onset of the bloom, El Niño climate conditions were occurring, with resultant cold sea temperatures off the north-east coast. Initially, GSM developed blood red guts, which was unappealing to consumers. This was due to a bloom of the ciliate *Mesodinium rubrum* (=*Myrionecta rubra*) which co-occurred with a *Noctiluca scintillans* (dinoflagellate) bloom. Shellfish were, however, unaffected at that time [143]. A *Karenia* bloom followed, developing during December 1992 [143,147] and BTXs were detected in shellfish from the area, although the *Karenia* species responsible for the toxins at that time has never been definitively determined [146]. It is interesting to note that using invertebrate larvae assays [91], *K. mikimotoi* was shown to impact pāua (abalone; *Haliotis iris*) larvae (LT_50_ 10 h at 12 × 10^6^ cells per litre), as pāua were reported to fall from the rocks (and mass mortalities of other marine organisms were recorded) during the early 1993 phase of the bloom. Low concentrations of brevetoxin-like lipid soluble toxins were reported in these affected shellfish [138].

A large bloom of *K. mikimotoi* in South Australia in 2014 caused massive mortality of abalone, rock lobster, turbo shells, various finfish, and echinoderms [95], and crude extracts of *K. mikimotoi* exhibited high lytic activity towards fish erythrocytes [92]. In NZ, *K. mikimotoi* has been implicated in fish mortalities [96,98]. In Northland, 2007, wild fish and eel mortalities were recorded, and an associated red discolouration of the seawater noted. *Karenia mikimotoi* was determined as the cause based on molecular assay results (fluorescent in situ hybridisation assays, quantitative PCR, and sandwich hybridisation assays). No BTXs were detected by liquid chromatography/mass spectroscopy and while haemolytic glycolipids could not be ruled out, anoxia was considered the prime cause of the mortalities [98].

Blooms of the fish-killing ***K. selliformis*** cause significant ecological damage including water column anoxia and marine fauna toxicity worldwide, including in NZ, Mexico, Tunisia, Kuwait, Iran, China, and Chile [112]. In mid-September 2021 over a period of two months, an unprecedented mixed bloom of *Karenia* species, including *K. selliformis*, *K. mikimotoi*, *K. longicanalis*, *Karlodinium* sp., and *Takayama* spp., occurred along the south-eastern coast of Hokkaido, Japan [148], with mass die-offs of sea urchins (*Strongylocentrotus intermedius* and *Mesocentrotus nudus*), salmon (*Oncorhynchus keta*) in fixed nets, octopus (*Paroctopus conispadiceus*), whelks (*Neptunea* spp.), chitons (*Cryptochiton stelleri*), bivalves (e.g., *Pseudocardium sachalinense*), and juvenile fish in rearing facilities [113,148]. Following laboratory exposures, adult Manila clams, *Ruditapes philippinarum*, have also been shown to be sensitive to exposure to *K. selliformis*, with cellular changes in the hemolymph recorded up to 6 weeks following exposure to bloom concentrations [103,115].

Extensive blooms of the then undescribed species, *K. selliformis*, occurred in Southland, NZ, during two successive summers 1993/94 and 1995/96 [142]. The toxin detected in extracts of exposed Bluff oysters, *Ostrea chilensis*, proved to be Gymnodimine and appeared to be a neuromuscular blocking agent, production of which was enhanced by addition of organic acids to the *K. selliformis* growth medium [149,150]. A south-north progression of the bloom, which also contained cells of *K. bicuneiformis* (as *K. digitata*) and *K. papilionacea*, was associated with the mortality of a variety fish and shellfish along the southeast coast [110,111]. Widespread deaths of pāua (*H. iris*) and a mass stranding, estimated at about one million tuatua (surf clams, *Paphies subtriangulata*) was reported. There was evidence that the bloom penetrated as far north as Port Underwood in Marlborough where it was associated with farmed juvenile GSM mortalities [110]. There was some experimental evidence that *K. selliformis* had a fast-acting toxic effect on oyster larvae [114].

The highly toxic ***K. brevisulcata*** (see [89]), which produces an array of toxins, including ten lipid soluble *K. brevisulcata* toxins and six water soluble brevisulcatic acids, killed all marine life in Wellington Harbour, NZ, in 1998 [90]. Tuna (*Thunnus alalunga*), striped marlin (*Kajikia audax*), and broad bill swordfish (*Xiphias gladius*), as well as kina/sea urchins (*Evichinus chloroticus*), starfish species, and pāua, were found washed up on the coastline and aerosols of the bloom caused respiratory distress and eye and skin irritation in humans in the vicinity [90]. Brevisulcatic acids extracted from *K. brevisulcata*, have been shown to be toxic to juvenile king salmon and snapper, *Chrysophrys auratus* (as *Pagrus auratus*) in experimental trials [91]. This toxicity extended to GSM, sea urchin and pāua larvae, and, to a lesser degree, sea slugs, *Pleurobranchaea maculata* and oyster (*C. gigas*) larvae [91].

***Karenia umbella*** was first associated with mortalities of cage-reared rainbow trout and Atlantic salmon in Tasmania in the late 1980s and early 2000s [116]. *Karenia umbella* is common in NZ’s coastal waters and blooms have been observed previously in sheltered embayments in the Marlborough Sounds. In early March 2018 and mid-February 2020, blooms of *K. umbella* were detected in Akaroa Harbour, south of Christchurch. The 2020 bloom dispersed throughout the harbour, including in and around several salmon farms in the area, causing distress to fish, although mortalities were low [135]. Samples collected from the salmon farm pens on 21st to 23rd February 2020 contained cell numbers up to 5 × 10^4^ cells L^−1^ [114]; however, toxin production by this species has not been confirmed.

### 2.3. Dinophysis spp.

Blooms of the thecate dinoflagellates *Dinophysis acuminata* and *D. acuta* have been documented throughout NZ, with the former occurring every year in an important GSM culture area, Port Underwood, in the Marlborough Sounds [151,152]. The cellular toxin content of bloom forming ***D. acuminata*** in NZ is low and the toxin profile is dominated by pectenotoxin-2 and dinophysistoxin-1 [152]. In Brazil, natural blooms of the same species have been shown to have negative effects on the immune system of adult *C. gigas*, the brown mussel, *Perna perna*, and the clam, *Anomalocardia brasiliana* (see [78,79]). Recent work by [80] in which gametes of *C. gigas* were exposed to *D. acuminata* for two hours, resulted in a reduction in fertilization success, which was attributed mainly to the presence of pectonotoxin-2. A similar species, *D. caudata*, which also produces pectenotoxins, has been shown to induce mucus and pseudofaeces production, paralysis, and negative effects on the digestive gland of adult scallops, *Patinopecten yessoensis*, and *Mimachlamys nobilis* (see [153]).

Although there have been no recorded fish mortality events associated with *Dinophysis* blooms, experimental exposure of fish to okadaic acid, dinophysistoxins, and *Dinophysis* cells produce a range of negative effects at different life stages, either by waterborne or dietary routes. These effects range from impacts on fish fitness, swimming performance, feeding, foraging, and escape from predators to reduced fish abundance [18]. There is a lack of information on lethal impacts, and sublethal and chronic effects of toxic *Dinophysis* in the environment [154].

### 2.4. Pseudo-nitzschia spp.

The amnesic shellfish poisoning diatom genus *Pseudo-nitzschia* is a regular bloom former throughout NZ coastal waters, particularly during the Austral spring and summer [155]. It is responsible for occasional domoic acid (DA) contamination of shellfish, in particular scallops, *Pecten novaezealandiae,* and, rarely, GSM. Many bloom-forming *Pseudo-nitzschia* spp. have been reported in NZ waters with highly variable DA production [155,156]. Dominant bloom formers have been identified as ***P. fraudulenta*** and ***P. pseudodelicatissima***, with ***P. australis*** and ***P. pungens*** common in spring, and ***P. multiseries***, ***P. multistriata***, ***P. heimii***, and ***P. delicatissima***, also detected in bloom concentrations at different times [25,155].

Effects on the feeding behaviour of bivalves have been observed in juvenile Pacific oysters, *C. gigas*, and scallops, *P. maximus*, exposed to French strains of *P. australis* and *P. fraudulenta*. Both bivalve species preferentially filtered non-toxic algae compared to the *Pseudo-nitzschia* cells and the clearance rate of *C. gigas* was reduced when exposed to toxic *P. australis* (see [47]). Interestingly, the presence of bivalves was found to induce an increase in the cellular DA contents of both *Pseudo-nitzschia* species [47]. Similarly, both juvenile and adult eastern oysters, *C. virginica*, and mussels, *Mytilis edulis*, were shown to reduce their clearance rate and increase pseudofaeces production when exposed to toxic *P. multiseries* [48,49,50,51]. The oysters did not differentiate between toxic *P. multiseries* and non-DA producing *P. delicatissima*, however, they exhibited comparable filtration of both algal species [50]. Physiological changes have also been observed in adult *C. gigas* exposed to DA-producing *P. multiseries*, with a spike in the hemocyte count and phagocytic activity of hemocytes recorded following only 4 h exposure [53]. The early life stages of *Pecten maximus* were found to be sensitive to exposure to dissolved DA with the growth, development to eye-spot stage and survival of larvae negatively affected [46]. However, the authors of [52] showed *P. multiseries* and DA exposure had no effect on the survival and development of *M. edulis* larvae.

In NZ, a novel, toxic, DA isomer, referred to as iso-DA-C, was detected in GSM, scallops and Pacific oysters harvested from the Marlborough Sounds and the Bay of Plenty in August 2001 [157]. The causative organism was determined as *P. australis*, but no effects were reported in the bivalves [158].

It is well documented that planktivorous fish can accumulate high levels of DA during toxic *Pseudo-nitzschia* blooms (e.g., [159]) and that DA can then be transferred up the food chain to seabirds and marine mammals when toxic fish are consumed [160]. While there are documented cases of resultant mass mortality events, there has been no environmental evidence for the fish being directly affected by toxic *Pseudo-nitzschia* blooms [161]. Direct injection of DA can cause excitotoxicity behaviours in fish, but ecologically relevant routes of exposure do not appear to cause harm to fish as they do to humans, marine mammals, and birds. Some effect may be induced by mechanical damage to gills including mucosa irritation (i.e., mucus overproduction), which can affect gill function or increase susceptibility to secondary bacterial, viral and parasitic infections [54].

### 2.5. The Raphidophytes: Heterosigma and Fibrocapsa

The first major loss of farmed fish in NZ occurred in January 1989 in Big Glory Bay, Rakiura/Stewart Island. More than eight hundred tonnes of Chinook salmon, valued at >NZ$17M at the time, died from impaired functioning of gills due to exposure to a bloom of the raphidophyte ***Heterosigma akashiwo*** (see [41,120], Figure 2). At the time, warmer than usual weather and a stable water column due to a La Niña phase of the Southern Oscillation provided ideal conditions for *H. akashiwo* to bloom, particularly as cells could migrate to deeper, more nutrient rich waters at night [41,120]. This species has been associated with fish kills globally [94,121,122,123] and since the first identification of *H. akashiwo* in NZ in 1989, it has been found to be common in NZ’s coastal waters and significant blooms have occasionally developed (for example, in Pelorus Sound, in the summer of 2017/18).

Both early and adult life stages of bivalves have been shown to be sensitive to bloom concentrations of *H. akashiwo*. The presence of algal cells reduced the swimming velocity of spermatozoa, as well as larval survival, activity, and metamorphosis of the Japanese Pearl oyster, *Pinctada fucata martensii*, and of the scallop, *Argopecten irradians*, with the toxic mechanism thought to be from the shed algal glycocalyx structures and mucus [124,125,126]. Adult Eastern oysters, *C. virginica*, exposed to bloom concentrations of *H. akashiwo*, closed their shells and reduced filtration [32,127] and in vitro exposure of *C. virginica* and hard clam (*M. mercenaria*) hemocytes to cultures of *H. akashiwo* resulted in increased haemocyte mortality [33].

In NZ, microalgal blooms are often associated with El Niño climate conditions and ***Fibrocapsa japonica*** was a major component of HABs during the early stages of the major *Karenia* bloom of 1993 [143,162]. The cells of *F. japonica* produce trichocysts and the ejection of these should be considered as a potential threat to shellfish larvae recruitment through entanglement [162]. Adult eastern oysters, *C. virginica*, exposed to water collected from a bloom of *F. japonica*, showed significantly increased lysosomal destabilization rates in the digestive gland [128] and other bivalve species may also be sensitive to exposure. Blooms of *F. japonica* have been implicated in the reduction of fish stocks overseas, but the mechanisms involved are still unclear. It has been demonstrated, however, that mortalities of the larvae of the common flat fish sole, *Solea solea*, occur in the presence of *F. japonica* cells, particularly when exposed to late exponential phase cultures, and that warmer temperatures had a distinct effect on the mortalities [129]. The involvement of endo- and exotoxins has been postulated with haemolytic polyunsaturated fatty acids as the main endotoxins, and other haemolysins and reactive oxygen species as the main exotoxins. Blooms of *F. japonica* have not been associated with fish mortalities in NZ, and studies of NZ strains did not detect ichthyotoxins [163].

### 2.6. Other Bloom Forming HAB Species in New Zealand

**Figure 3:**
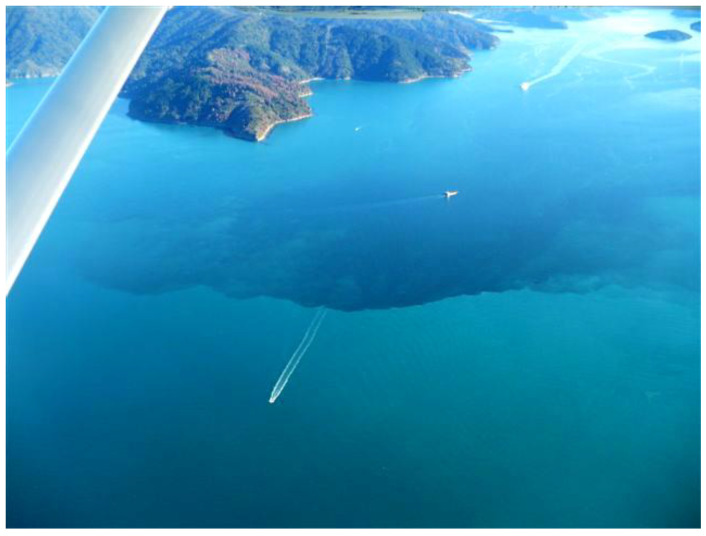
The 2010 *Pseudochattonella* bloom, dominated by *P. verruculosa*, present in Queen Charlotte Sound, Marlborough sounds, New Zealand. Credit: Lincoln MacKenzie.

In 2010, approximately two hundred tonnes of farmed Chinook salmon died in Queen Charlotte Sound, Marlborough Sounds. The cause proved to be the dictyophyte ***Pseudochattonella verruculosa*** (Figure 2 and Figure 3) and the mortalities occurred at cell concentrations ten-fold less than those caused by the earlier *H. akashiwo* bloom in Big Glory Bay. The pathologies of the dead fish were inconclusive, although hypoxia was considered a primary cause. Moving net pens to a bloom-free site saw the recovery of the surviving fish. The bloom occurred after a long period of heavy rainfall and when seawater temperatures were at their annual minimum (12 °C), as was daylength, at nine hours [43].

Highly potent palytoxin-like compounds may be produced by the thecate dinoflagellate genus *Ostreopsis*. In NZ, ***Ostreopsis* cf. *siamensis*** (=*Ostreopsis* sp.) reported by [164] produces such compounds [165,166] and kina (sea urchin, *Evechinus chloroticus*), are particularly vulnerable to the toxins. During recurrent blooms of *O.* cf. *siamensis* in Northland waters, mass mortalities occurred [117], Figure 2. Declines in adult kina density in the order of 56–60% were reported following a period of calm sea conditions with warmer than average water temperatures [118]. Larvae were, however, unaffected by *O.* cf. *siamensis* in in vitro assays, suggesting that the survival and recruitment of larvae in the wild may continue despite blooms [166].

On occasion, reports of blood red oysters are received [167] and this can usually be traced back to blooms of the deeply red coloured ciliate ***Mesodinium rubrum*** in the vicinity. The oysters are generally considered unfit to eat but are not toxic and appear unaffected by the ciliate.

In 1983, in Northland’s Bream Bay, NZ, fish and shellfish mortalities were associated with a mixed bloom of the diatom *Cerataulina pelagica* (Figure 2) and an undescribed haptophyte, a *Prymnesium* species. This genus had been associated with fish mortalities previously. The new species was described and classified as ***P. calathiferum*** (see [119], Figure 2) and proved closely related to the biotoxin producer *P. parvum*. The latter produces prymnesins and has caused mortalities of fish and invertebrates world-wide (see [168,169]).

Even when HABs are non-toxic, they may cause shellfish or finfish mortalities due to a lack of oxygen, particularly when they occur in confined bays and harbours. For example, the usually harmless green flagellate, ***Tetraselmis* sp**., caused the death of pilchards, *Sardinops sagax* in a Wellington Harbour lagoon in 1993 due to an oxygen deficit [170]. The diatom ***Cerataulina pelagica*** similarly caused finfish and shellfish deaths, in Bream Bay in 1982 [76]. Blooms have been reported to cause clogging of gills through production of mucus for more than a century. ***Gonyaulax fragilis*** produces transparent exo-polymers composed primarily of galactose and glucose monomers. This ‘slime’ can cause mass mortalities of marine fauna and impede fishing activities and has been responsible for fish deaths in Tasman Bay, NZ [25,77], Figure 2. Ubiquitous spiny diatoms such as ***Chaetoceros* spp**. can cause mechanical damage due to the barbed setae becoming embedded in the lamellar epithelium of salmonid gills with resultant production of excessive amounts of mucus. Death can result from asphyxia or increased susceptibility to secondary infections [171,172].

## 3. Future Issues

The following section (and Table 2) details HAB species that, although they have not yet bloomed, occur in NZ waters, and, therefore, have a potential to harm marine life. This section includes monitoring data from aquaculture production areas around NZ (Section 3.1.1). Section 3.2 discusses HAB species that are likely to be encountered in NZ in the future.

### 3.1. Ichthyotoxic HAB Species Present in New Zealand

The spirolide producing dinoflagellate, ***Alexandrium ostenfeldii***, has been shown to negatively affect commercially important bivalve species [173,174]. Cysts of *A. ostenfeldii* are common in coastal sediments around NZ but are rarely found in the plankton [45]. These resting cyst beds may indicate past blooms [110] or that *A. ostenfeldii* is mainly a benthic species in NZ.

The karlotoxin producing nonthecate dinoflagellate ***Karlodinium veneficum*** (previously *K. micrum*) has been recorded in both the northeastern Hauraki Gulf and the more southern Marlborough Sounds [202]. This species has been shown to have harmful effects on shellfish, including mussels, *M. edulis*; clams, *Mercenaria mercenaria*; and oysters, *Crassostrea virginica* and *C. ariakensis* (see [185,186,187,188]). This ichthyotoxic species was implicated in massive finfish mortalities (more than 100,000 fish) in the sub-tropical Upper Swan River Estuary in Western Australia [182]. Numerous fish species were affected, including black bream, *Acanthopagrus butcheri*; Perth herring, *Elops machnata*; and Swan River gobies, *Pseudogobius olorum*. Gill histopathology was comparable to the damage shown in laboratory experiments, and it was also demonstrated that microalgal cell lysis was required for the toxic effects. In the USA, *Karlodinium veneficum* (as *K. micrum*) has been implicated in three separate fish kills of hybrid striped bass (*M. chrysops × M. saxatilis*) in Chesapeake Bay, Maryland [183,184] and toxic substances were detected in cell-based assays. The toxins, karlotoxins, have been characterised and it is possible that they are involved in predation by the mixotrophic dinoflagellates [203]. Hong Kong, and the wider coastal waters of south China, were impacted by a massive bloom of the related *K. digitatum* (previously *K. digitata*) in 1998, with concurrent fish kills. The bloom was indiscriminate, killing at least twenty-two fish species, both farmed (caged) and naturally occurring. Low wind speeds, warming harbour waters, and high nitrogen and phosphate levels appeared to trigger the bloom [204]. The genus *Karlodinium* should, therefore, be of concern for finfish aquaculture and requires further research in NZ as well as regular monitoring. It is plausible that the genus is quite diverse with a single transect of the Southern Ocean south of Tasmania, Australia, giving rise to five new *Karlodinium* species [205].

In Japan, ‘red tides’ of the thecate dinoflagellate ***Heterocapsa circularisquama*** devastated the bivalve shellfish industry in 1998, impacting on the mariculture of the Japanese pearl oyster, *Pinctada fucata martensii* [178]. Only invertebrates were affected at that time, and the cause appeared to be a labile protein-like complex on the cell surface which was causing the detrimental effect on bivalves. Further studies revealed mortalities in several commercially important bivalve species exposed to this microalga [29,85,179,180]. Five *Heterocapsa* species have been recorded in NZ, including *H.* cf. *circularisquama,* which occurs in northern sub-tropical waters. To date, no bivalve mortalities have been reported, although *H. illdefina* has been responsible for ‘swimmers itch’ and may therefore have some toxic properties [181].

The thecate dinoflagellate ***Prorocentrum rhathymum*** (syn. *P. mexicanum* under investigation) has been recorded in NZ [206], but has not been associated with marine mortalities. It has, however, been linked to oyster spat, *C. gigas* mortalities in Tasmania, Australia. Intraperitoneal mouse bioassays revealed fast acting toxins in methanol but not aqueous extracts of *P. rhathymum*, with mice dying in less than 20 min [197].

Prevalent along southeast Asian and north American coasts, the thecate dinoflagellate ***Cochlodinium polykrikoides*** (syn. *Margalefidinium polykrikoides*) has been responsible for massive fish kills in those regions (e.g., [94,175,176,177]). The dinoflagellate species poses a potential risk to finfish in NZ as it has been reported in NZ waters and would most likely bloom in offshore northern waters where temperature ‘hot spots’ have occurred [207,208].

The globally distributed, heterotrophic, dinoflagellate genera ***Pfiesteria*** and ***Pseudopfiesteria*** are both considered responsible for fish kills in eutrophic estuaries in the USA [191], although considerable strain variability in toxicity has been noted [209]. These dinoflagellates possess a peduncle (feeding tube) that can be extended between its sulcal plates and used to feed on micro-algae, bacteria, fish, and other organisms. This feeding behaviour is known as myzocytosis and can cause tissue damage and contribute to the death of fish [191,192] and bivalve larvae [193,194].

Both *Pfiesteria* and *Pseudopfiesteria* have been reported in NZ, particularly in estuarine habitats, and the potential risk to animal health is dependent on the continued control of nutrient inputs into NZ’s estuaries and brackish lakes. The thecate *Pfiesteria piscicida* and *Ps. shumwayae* are resident in the sediments of Tasman Bay’s well-flushed estuaries and Canterbury’s brackish lakes, as determined by quantitative PCR assays targeted at ribosomal DNA [195,210,211] and confirmed by scanning electron microscopy of motile cells [195]. Most detections have been of cysts in sediments, but motile cells have been detected in water samples in late summer–autumn [195]. Similarly, *Ps. shumwayae* causes mortality of larval and adult bivalve species [196] and a strain of *Ps. shumwayae,* isolated from Tasman Bay sediments, has been shown to be ichthyotoxic [195].

The raphidophyte genus ***Chattonella*** has caused massive fish kills in Australia. Mass mortalities of caged bluefin tuna, *Thunnus maccoyii*, in South Australia in 1996 were caused by *C. marina* blooms with a cost to the industry of an estimated AU$45 million [200,201]. The higher potency of the Australian blooms (which killed at 66,000 cells L^−1^ compared to Japan at 500,000 cells L^−1^ where similar mass fish mortalities have occurred; see [199]) was attributed to the higher sensitivities of tuna and to the higher ichthyotoxicity of Australian high-light adapted algal strains [212]. The toxic potential of *Chattonella* is believed to be associated with a high production of reactive oxygen species [213]. Early life stages of the Japanese Pearl oyster, *Pinctada fucata martensii* have been shown to be sensitive to *C. marina* with negative effects observed on sperm swimming velocity [124] and on the umbo and pre-settling larval stages [125]. In NZ, ***C. marina* var. *antiqua*** has been isolated from the northeast and the southernmost coast of the North Island. Cultures were tested and were negative for BTXs [214].

**Figure 4:**
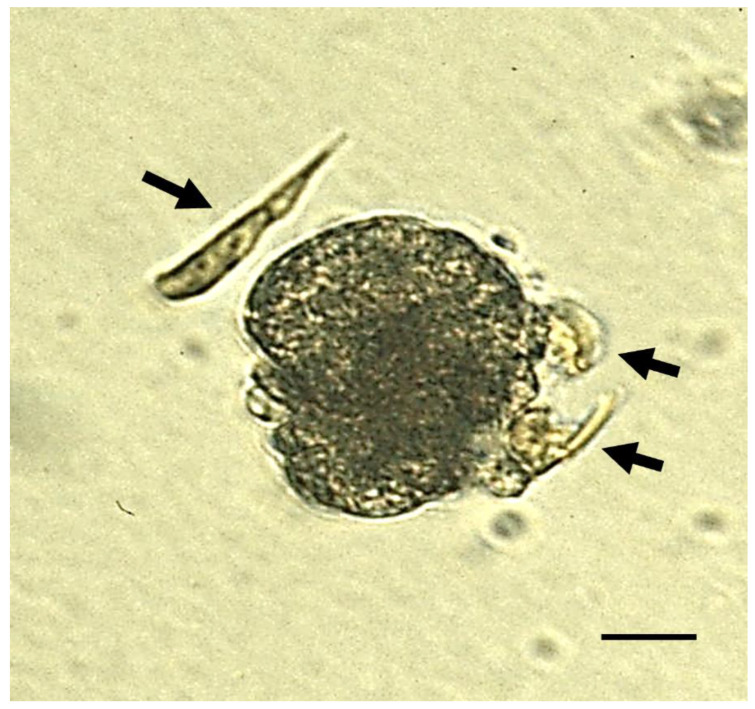
Cells of *Pavlomulina* (black arrows): swimming freely (top left) attached to trochophore larvae of *C. gigas* (right) and swelling (top right). Scale bar = 20 µm. Photo credit: Othmand Bojo.

Another potentially problematic microalga is the newly described haptophyte species ***Pavlomulina ranunculiformis*** (Figure 4), which has been observed attaching to oyster larvae and then swelling (apparently becoming engorged) before dropping away [28]. Whether this proves to be an issue for aquaculture remains to be seen.

#### 3.1.1. Potentially Harmful Taxa Detected Using High-Throughput Sequencing Metabarcoding

Seawater samples from around the South Island of New Zealand, collected as part of the New Zealand Marine Biotoxin Monitoring Programmes and containing cells of potentially ichthyotoxic species were analysed using high-throughput sequencing metabarcoding. Four primer sets (Supplementary Table S1) targeting the 18S and 28S ribosomal RNA regions were used to attempt to capture a wide range of potentially toxic taxa. Specific primers for dinoflagellates and haptophytes were used to maximise coverage of these important groups. Morphological classification of these groups is also problematic, and we aimed to increase molecular data and knowledge of these harmful taxa. No raphidophyte or dictyochophyte taxa were detected using any of the primer pairs and so our analyses focused on diatoms, dinoflagellates, and haptophytes.

**Figure 5:**
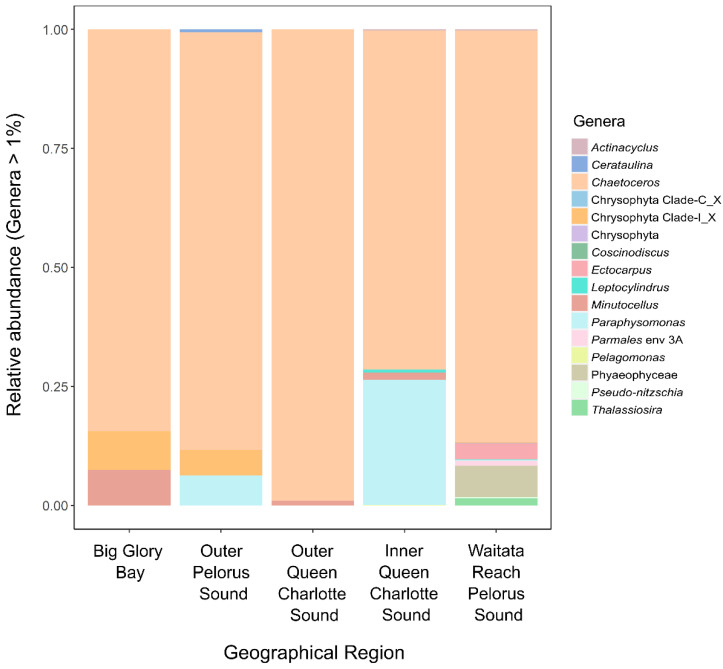
Relative abundance of dominant diatom and other eukaryotic phytoplankton (excluding dinoflagellate and haptophytes) genera from each of the sampled geographic regions amplified using universal 18S ribosomal RNA primers. No raphidophytes or dictyochophytes were detected. X = Unclassified from the higher rank of taxonomic classification.

All sites were dominated by the diatoms *Chaetoceros* spp. (Figure 5), and the harmful species *C. convolutes* was detected (Supplementary Table S2). This species has large barbs which can cause finfish mortalities even at very low concentrations due to microbial infections of damaged gill tissue, haemorrhage of gill capillaries or suffocation from excess mucus production at the sites of penetration of the gills by the spines [171]. *Pseudo-nitzschia pseudodelicatissima* was also found at Waitata Reach (Figure 5). It is a common bloom forming species in New Zealand which can also cause physical damage to fish gills.

**Figure 6:**
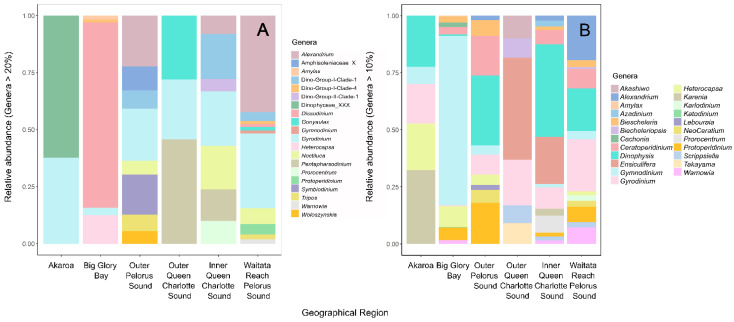
Relative abundance of dominant dinoflagellate genera from each of the sampled geographic regions amplified using (**A**) Universal 18S ribosomal RNA primers and (**B**) Dinoflagellate specific 28S ribosomal RNA primers. X = Unclassified from the higher rank of taxonomic classification.

The diversity of dinoflagellates at all sites was high for both gene regions used, and several harmful taxa were detected, including *Alexandrium* spp., *Gonyaulax fragilis*, *Dinophysis* spp., *Karenia* spp., *Karlodinium* spp., *Pfiesteria* sp., and *Prorocentrum* spp. (Figure 6). Most of these genera were present at low levels; however, *Alexandrium pacifium* was a dominant species at most of the Marlborough Sounds sites. A bloom of *Karenia umbella* was detected in Akaroa (Figure 6B), a species that has caused issues in this harbour previously. *Karlodinium* and *Takayama* species were also both detected and while no associated fish kills or toxin events have occurred in New Zealand to date, both genera have been implicated in fish kills in Australia [111].

Some genera, such as *Dinophysis* and most *Prorocentrum* spp., were unable to be classified to species-level using the two gene regions used, although good resolution was achieved for other genera including *Alexandrium*, *Karenia*, and *Karlodinium*, highlighting the usefulness of these gene regions for nucleic acid-based detection methods [215].

**Figure 7:**
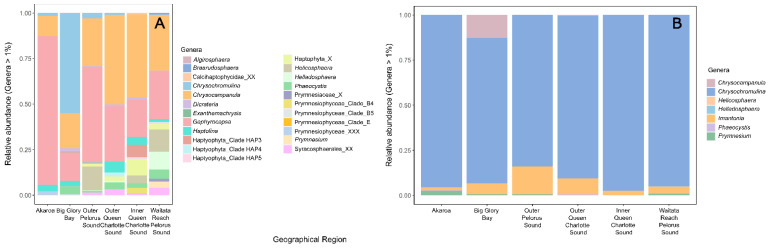
Relative abundance of dominant haptophyte genera from each of the sampled geographic regions amplified using (**A**) Haptophyte specific 18S ribosomal RNA primers and (**B**) Haptophyte specific 28S ribosomal RNA primers. X = Unclassified from the higher rank of taxonomic classification.

The harmful genus *Chrysochromulina* was common at all sampled sites (Figure 7), although the most common species associated with fish mortalities worldwide, *C. polylepis* and *C. leadbeateri*, were not detected (Supplementary Table S2). Both gene regions showed good taxonomic resolution for this genus. The 18S ribosomal gene did detect a higher number of haptophyte species, likely due to a more comprehensive database available for this region. Other harmful genera detected were *Phaeocystis* and *Prymnesium*, although these could not be resolved to the species level (Figure 7; Supplementary Table S2).

These data show that many potentially harmful taxa are common at important aquaculture sites in New Zealand, especially in the important aquaculture region, the Marlborough Sounds. An increase in the DNA sequence data available for these taxa will enable the development of more specific and rapid molecular tools for the detection and characterisation of HABs. This is especially important for species that are difficult to identify by light microscopy (e.g., *Chrysochromulina*; [216]).

### 3.2. HABs in a Changing Environment

Sea surface and sub-surface waters around New Zealand are warming [207]. From 2002 to 2020, there was an estimated 0.2–0.4 °C decade^−1^ increase in sea surface temperature in the important GSM-producing Pelorus Sound, Marlborough Sounds, which has likely contributed to difficulties in gaining year-round wild-spat supply and a decrease in mussel condition, leading to a reduction in the value of harvest [217]. In addition to this ocean warming, the 2017 marine heatwave, that persisted for the entire Austral summer, during which sea surface temperature abnormalities reached up to +3.7 °C in the Eastern Tasman Sea [208], contributed to the deaths of many salmon in the Marlborough Sounds [39].

**Figure 8:**
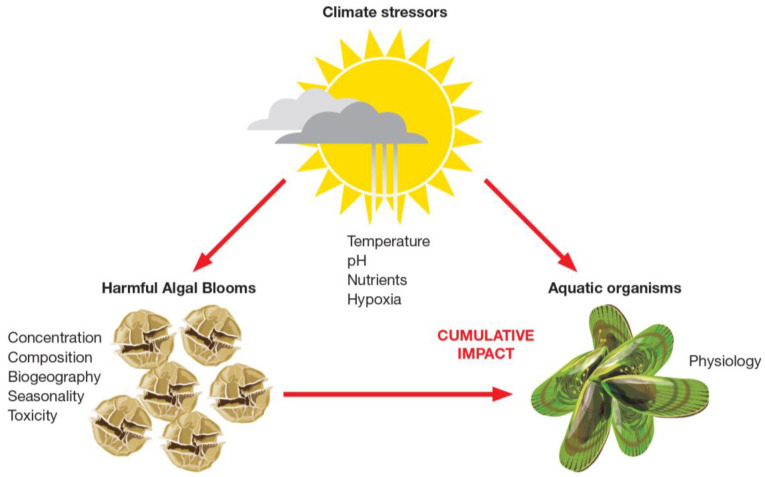
The effects of climate change stressors (e.g., temperature, pH, nutrients, hypoxia, salinity, turbidity, and anthropogenic) on Harmful Algal Blooms (HABs) and fish and shellfish species. Alterations in HAB concentration, species composition, biogeography, seasonality, and toxicity in a changing environment, combined with an altered physiological state of fish and shellfish species, could results in antagonistic, additive or synergistic effects on commercially important species. Image: Eden Cartwright, Bird Circus.com.

Increased temperatures and temperature anomalies affect the metabolic and physiological responses of commercially important fish and shellfish species, rendering them more susceptible to the effects of abiotic and biotic stressors such as HABs (Figure 8, [218,219,220]). Previous studies have demonstrated the interactive effects of harmful algae and other biotic stressors (i.e., infectious agents), on the immune response of bivalves [221,222]. Research on the multiple stressor effects of temperature changes (and related climate change stressors) and HABs is lacking. This knowledge is essential to obtain a more ecologically accurate assessment of the effects of HABs on commercially important species [223].

Increased water temperatures, temperature anomalies, and temperature-driven stratification will also change the abundance, composition, biogeography, and seasonal distribution of the phytoplankton (Figure 8, [5,224]). Such changes may result in exceptional HABs, such as the unprecedented large-scale HABs reported in the coastal waters off the south-eastern coast of Hokkaido, Japan, from mid-September 2021, about one month after intense and extensive marine heatwaves subsided [113].

The toxicity of HABs is already known to vary significantly among isolates of the same algal species [225] and even within strains from the same population [226]. Climate-change driven changes in the phytoplankton may also change HAB toxicity, further highlighting the need to investigate the effects of local, recent, HAB strains of interest on commercially important species. The use of relevant bioassays, using bivalve gametes [22,74], larvae [12,22] hemocytes [33], and fish gill cells [227], for example, will allow rapid screening of potentially toxic HAB species and determination of what is causing the toxic effects (i.e., known toxins or other, often uncharacterised, bioactive compounds), enabling refinement of risk assessments and accurate mitigation strategies for producers.

Changes in the phytoplankton may also lead to the proliferation of HABs novel to NZ. The ciguatera fish poisoning dinoflagellate genus, *Gambierdiscus*, has been reported in New Zealand coastal waters and is likely to proliferate with warming waters [228]. The dinoflagellate, *Alexandrium catenella* (=*A. fundyense*, Group I, [131]) has contaminated shellfish in Australia, particularly since 2012 [25,229], and has been responsible for mass shellfish and fish kills world-wide (e.g., [230,231,232]). It is likely *A. catenella* is present in offshore waters of NZ, given the proximity to Tasmania, Australia. As is hypothesised in Australia, persistent ocean stratification driven by climate change, may well lead to the emergence of blooms of this species in NZ [25], as has been seen with *A. pacificum* with its spread to the top of the South Island [233]. The use of molecular methods for species detection and comprehensive monitoring programmes will ensure that such changes are identified promptly.

## 4. Conclusions and Future Directions

Harmful algal blooms (HABs) have wide-ranging effects on aquatic species of commercial importance. In addition to the lethal effects of HAB exposure, sublethal effects can also reduce commercial productivity by reducing the fitness of the animal [154]. The use of molecular methods and comprehensive HAB monitoring programmes allow for early detection of novel and problem species [234] and use of cost-effective, high through-put bioassays can be used to rapidly identify any local, novel HAB species of concern. Moreover, improvements in predicting the movement and size of HABs, such as the use of statistical and machine learning HAB forecasting tools (see [6,235]), are enabling more proactive management responses. Mitigating the impacts of HABs on industry, however, is challenging. Stock management strategies could include informed site selection, the use of physical barriers such as skirts around fish net pens, emergency or early harvest, or moving stock to offshore areas [6,234]. Novel technologies, such as floating closed containment systems, are driving innovation in aquaculture system design given they may offer protection against HABs as well as disease and temperature [236]. Alternatively, land-based recirculating aquaculture systems offer ultimate control in water quality for high-value aquaculture species. There remain, however, large knowledge gaps on the effects of HABs, notably in conjunction with increased temperature (and other climate change stressors) and research should focus on the effects of these and other relevant multiple stressors.

## 5. Materials and Methods

### 5.1. Sampling, PCR Conditions and High-Throughput Sequencing

Seawater samples (100 mL) were collected from around the South Island of New Zealand as part of the New Zealand Marine Phytoplankton Monitoring Programme during routine weekly phytoplankton monitoring. Samples containing cells of potentially ichthyotoxic species (as determined by the Cawthron Phytoplankton Monitoring Laboratory by light microscopy analysis) were filtered (Durapore membrane filters, 0.45 μm, Millipore, Bedford, OH, USA) and stored at −20 °C until DNA extraction. Genomic DNA was extracted using DNeasy PowerSoil isolation kits (Qiagen, Valencia, CA, USA) following the manufacturer’s instructions using an automated homogenizer (1600 MiniG Automated Tissue Homogenizer and Cell Lyser, SPEX SamplePrep, Metuchen, NJ, USA) and a robotic workstation for DNA extraction (QIAcube, Qiagen). Negative extraction controls were performed every 23 samples. For each sample, gene regions were amplified by Polymerase Chain Reaction (PCR), using the primers list in Supplementary Table S1 The primers were modified to include Illumina^TM^ overhang adaptors following the dual-indexing method described in [237]. All PCR reactions were undertaken in duplicates with 450 nM of each primer, 13 µL of 2× MyFi™ Mix (Bioline, London, UK), ca. 5 ng of DNA, and sterile water for a total reaction volume of 25 µL. Cycling conditions were: 95 °C for 5 min, followed by 32 cycles of 95 °C for 30 s, 54 °C for 30 s, 72 °C for 45 s, and a final extension of 72 °C for 7 min for the 18S and 28S regions; and 98 °C for 30 s, followed by 35 cycles of 98 °C for 10 s, 58 °C for 30 s, 72 °C for 30 s, and a final extension of 72 °C for 10 min for the 18S haptophytes and 28S haptophytes target regions. Duplicates of PCR products were pooled and visualized on 1.5% agarose gel with Red Safe™ DNA Loading Dye (Herogen Biotech) and UV illumination. PCR negatives were run to assess for contamination during the PCR steps. The PCR products were purified, cleaned of primer dimers, and normalized using SequalPrep Normalization plate (ThermoFisher, Waltham, MA, USA), and submitted to Auckland Genomics (University of Auckland, New Zealand) for library preparation. Sequencing adapters and sample-specific indices were added to each amplicon via a second round of PCR using the Nextera™ Index kit (Illumina Inc., San Diego, CA, USA). Amplicons were pooled into a single library and paired-end sequences (2 × 250 bp) generated on a MiSeq^®^ instrument. The sequencing libraries were prepared following the Illumina 16S Metagenomics Library Prep manual. Quality control was undertaken using a bioanalyzer before the library was diluted to 4 nM and denatured. A 15% PhiX spike was used, and the final loading concentration was 7 pM. Sequence data were automatically demultiplexed using MiSeqR Reporter (version 2, Illumina Inc.), and forward and reverse reads assigned to samples. Raw sequence reads were deposited in the National Center for Biotechnology Information (NCBI) short read archive under the accession number PRJNAXX.

### 5.2. Amplicon Sequence Variant Inference and Taxonomic Assignments

Bioinformatic pipelines for all of the rRNA genes were identical unless otherwise stated. Raw reads were processed, after primers being removed with cutadapt [238], using the DADA2 package [239] within R. Reads were truncated to 228 and 230 bp and filtered with a maxEE (maximum number of “expected errors”) of 2 and 4 for forward and reverse reads, respectively (reads not reaching this threshold were discarded). DADA2 constructs a parametric error matrix (based on the first 108 bps in the dataset), the samples are dereplicated and sequence variants for the forward and reverse reads are inferred based on the derived error profiles from the samples. Singletons observed in the inference step are discarded. Subsequently, paired-end reads were merged with a maximum mismatch of 1 bp and a required minimum overlap of 10 bp. Forward and reverse reads, which did not merge were not included in further analysis. Chimeras were removed using the function removeBimeraDenovo. The resulting chimera-checked, merged amplicon sequence variants (ASVs) were used for taxonomic classification using the PR2 database [240] for the 18S and the 18S haptophytes datasets, the LSU database [241] for the 28S dataset, and the LSU Haptophyta database [242] for the 28S haptophytes dataset. The sequences were classified based on the rdp classifier [243] with a bootstrap of 50 to be able to get classifications at higher taxonomic levels. The results were parsed into a table using the phyloseq package [244], and negative controls were assessed and the sum of reads from contaminating ASVs was subtracted from the samples. For comparisons between samples, subsampling to an even depth was undertaken for each sample at a depth of 10,000 reads for the 18S and 28S datasets, and 4000 reads for the smaller 18S haptophytes and 28S haptophytes datasets.

Stacked bar plots and taxonomy tables were generated using the package ggplot2 [245] in R based on the average relative abundance of sequence reads attributed to a given taxonomy at each location sites.

## Figures and Tables

**Table 1:** Harmful algal bloom species which have bloomed in New Zealand (NZ) waters and their known effects on commercially important shellfish and fish species. NZ refs are in bold.

Class	Genus	Species #	Effects on Shellfish and Fish	Exposure to	Key References
**Bacillariophyceae**	*Pseudo-nitzschia*		Larvae of *Pecten maxiumus:* growth, development and survival reduced	Domoic Acid (DA)	[46]
		*P. australis* (DA-producer) *P. fraudulenta* (DA-producer)	Juvenile *P. maximus* & *Crassostrea gigas*: Preferential filtration of non-toxic algae *C.g.*: Reduction in clearance rate (*P. australis* only)	Whole cell culture	[47]
		*P. multiseries* (DA-producer)	Juvenile & adult *C. virginica*: increased pseudofaeces production, lower filtration rates	Whole cell culture	[48,49,50]
			*C.v.* & *Mytilus edulis*: selective rejection of toxic cells	Whole cell culture	[51]
			*M.e*.: no effect on larval survival & development but, increased phenoloxidase production	Whole cell culture & DA	[52]
			Adult *C. gigas*: Reduction in the number and phagocytic activity of hemocytes	Whole cell culture	[53]
		*P. delicatissima* (non-toxic)	Adult *C.v*.: lower filtration rate	Whole cell culture	[50]
			Juvenile sea bass (*Dicentrarchus labrax*): mucus over production in gills	Whole cell culture	[54]
**Dictyophyceae**	*Pseudochattonella*	*P. verruculosa* (Hara & Chihara)	Mortality of sea-cage salmon, *Oncorhynchus tshawytscha*	Field bloom	[43]
**Dinophyceae**	*Alexandrium*		Adult and larvae of various fish spp.: mortalities and impairment of sensory-motor function	Field blooms, whole cell culture, paralytic shellfish toxins & saxitoxins	[19] (and references there in)
		*Alexandrium pacificum*	Adult *Perna canaliculus:* Reduction in byssus production, reversable temporary increase in oxygen uptake	Whole cell culture	[55,56] (as *A. tamarense*)
			*P.c.* spat (2 mm): Reduction in byssal pad production and growth	Whole cell culture	[57]
			Adult *Pecten novaezelandiae:* clearance rate reduced and altered in other bivalve species.	Whole cell culture	[58,59] (as *A. tamarense*)
			Adult *Argopecten irradians*, *Geukensia demissa*, *Mercenaria mercenaria*, *Mya arenaria*, *Mytilus edulis*, *Ostrea edulis*, *Paphies donacina*, *Placopecten magellanicus* and *Spisula solidissima:* various effects including shell valve closure, changes in clearance rate, production of mucus, inhibition of byssus production, violent swimming & erratic siphon activity	Whole cell culture	[34,55,60,61,62] (as *Gonyaulax tamarensis, Protogonyaulax tamarensis* and *A. tamarense*)
			Juvenile *C. gigas*: changes in hemocyte parameters	Whole cell culture	[63] (as *A. catenella*)
			*A. irradians* larvae: increased mortalities; reduced activity, and growth of larvae; reduced attachment and climbing ability of juveniles	Whole cell culture & saxitoxin	[64,65] (as *A. tamarense,* strain ATHK)
		*Alexandrium minutum*	Adult *C. gigas*: reduced clearance and filtration rate, increased shell micro-closures, inflammatory response in digestive gland, increased circulating hemocyte concentration and phagocytic ability, genotoxic effects and increased detoxification/antioxidant gene expression. Reduced motility and ATP content of sperm, smaller larval size and increased mortalities at settlement	Whole cell culture	[66,67,68,69,70,71,72,73]
			Gametes and larvae of *C.g*.: increased ROS in oocytes, decrease in sperm viability & fertilization. Reduced larval hatching, swimming, feeding, growth, settlement and survival	Whole cell culture	[16,17,74]
			Adult *P. maximus*: delayed shell growth, alteration of sensing processes, less efficient escape response, muscular damage	Whole cell culture	[75]
	*Cerataulina pelagica*	*C. pelagica*	Mortality of Fin fish & shellfish	Field bloom (lack of oxygen)	[76]
	*Gonyaulax*	*Gonyaulax fragilis*	Mortality of marine fauna including fin-fish.	Field bloom (slime)	[77]
	*Dinophysis*		Lethal and sub-lethal effects on various fin fish species, including: behavioural changes, poor co-ordination, inactivity, oxidative stress and histological changes in adults, and: reduced hatching, swimming activity, growth & feeding of larvae	Okadaic Acid & dinophysis toxins	[18] (and references there in)
		*Dinophysis acuminata*	Adult *C. gigas*, *Perna perna* & *Anomalocardia brasiliana:* Changes in the hemocyte immunological parameters, especially in *P. perna*. Hemocyte infiltration in the digestive gland of *C. gigas*.	Field bloom	[78,79]
			Gametes of *C.g.:* increased oocyte mortality & reduced fertilization success	Whole cell culture	[80]
	*Gymnodinium*	*Gymnodinium catenatum*	Mortality of finfish	Field blooms	[81,82]
			*C. gigas* spat (3 mm): reduced clearance rate, increased valve closure & pseudofaeces production. Inflammation of the gill and digestive gland	Whole cell culture	[83,84]
			*C.g.* larvae: no observable effects	Whole cell culture	[85]
			Juvenile *Argopecten ventricosus* & *Nodipecten subnodosus*	Whole cell culture	[86,87,88]
	*Karenia*	*K. brevisulcatum* (Chang) Gert Hansen & Moestrup	Mortalities of fish and shellfish	Field blooms	[89,90]
			Mortality of various larval & juvenile fish and shellfish species (*Oncorhynchus tschawytscha*, *Chrysophrys auratus*, *P. canaliculus*, *Evechinus chloroticus* and *Haliotis iris*, *Pleurobranchia maculata*, *C. gigas*)	Whole cell culture, cell free culture, SPE extract, purified brevisulcatic acids	[91]
		*K. mikimotoi* (Miyake & Kominami ex Oda) Gert Hansen & Moestrup	Fish (*Sciaenops ocellatus*) erythrocytes: hemolytic activity	Crude algal extract	[92]
			Lethal and sublethal effects on finfish and shellfish	Field blooms, whole cell culture	[23,93,94,95] (and references there in)
			Fin fish, eel and abalone mortalities	Field blooms	[96,97,98]
			Various adult shellfish species: reduced clearance rates, changes in immune functions, reduced escape locomotion and paralysis	Field blooms, whole cell culture	[33,61,93,99,100,101,102,103,104] (as *Gyrodinium aureolum*)
			Various larval shellfish species: embryo, larval and spat mortalities, reduction in activity rate	Whole cell culture, SPE extract, bloom water, filtered bloom water	[85,91,105,106,107,108,109]
		*K. sellifomis* Haywood, Steidinger & MacKenzie	Mortality of various fin fish & shellfish, especially *Haliotis iris*, *Paphies subtriangulata* & *P. canaliculus*	Field bloom	[110,111]
			Mortality of various fin fish & shellfish	Field bloom	[112,113] (references there in)
			*C. gigas* larvae: mortalities	Whole cell culture	[114]
			Adult *Ruditapes philippinarum*: hemocyte variables changed	Whole cell culture	[103,115]
		*K. umbella* de Salas, Bolch & Hallegraeff	Mortalities of *Oncorhynchus mykiss* & *Salmo salar*	Field bloom	[116]
	*Ostreopsis*	*O.* cf. *siamensis*	Mortalities of sea urchins, *Evechinus chloroticus*	Field bloom	[117,118]
**Haptophyceae**	*Prymnesium*	*P. calathiferum* Chang & Ryan	Fish and shellfish mortalities	Field bloom	[119]
**Raphidophyceae**	*Heterosigma*	*H. akashiwo* (Hada) Sournia	Mortality of *O. tschawytscha*	Field bloom	[41,120]
			Mortalities of various juvenile and adult fin fish	Field blooms, toxins from blooms	[94,121,122,123]
			*Pinctada fucata martensii* & *Argopecten irradians* gametes & larvae: Reduced sperm swimming velocity, increased mortalities & abnormalities, reduced activity of trocophore & D-larvae	Cell free culture, whole cell culture	[124,125,126]
			Adult *C. virginica*: shell closure, reduction in filtration and increased hepatopancreas lysosomal destabilization	Whole cell culture	[32,127]
			*C. virginica* and *M. mercenaria* hemocytes (in vitro): mortality	Whole cell culture, culture filtrate	[33]
	*Fibrocapsa*	*F. japonica*	Adult *C. virginica:* increased lysosomal destabilization in digestive gland	Bloom water	[128]
			*Solea solea* larvae: mortality	Whole cell culture, culture extracts	[129]

#: Species classifications based on AlgaeBase (Guiry in [130]). *Alexandrium* classification as for [131]. *A. catenella* (=*A. fundyense*).

**Table 2:** Harmful algal bloom species which have been detected but not bloomed in New Zealand waters and their known effects on commercially important shellfish and fish species. NZ references are in bold.

Class	Genus	Species #	Effects on Shellfish and Fish	Exposure to	Key References
**Dinophyceae**	*Alexandrium*	*A. ostenfeldii*	Adult *Ruditapes philippinarum* & *C. gigas*: tissue inflammatory response, changes in hemocyte morphology, oxidative stress response in the gills	Whole cell culture	[173,174]
	*Cochlodinium (=Margalefidinium)*	*C. polykrikoides*	Mortalities of finfish	Field blooms	[94,175]
			Adult *Argopecten irradians*, *C. gigas*, *M. mercenaria:* mortalities and reduced growth	Field blooms	[176] (and references there in)
			Juvenile *A. irradians* and *Cyprinodon variegates*: mortalities	Whole cell culture, cell free culture medium	[177]
	*Heterocapsa*	*H.* cf. *circularisquama* Horiguchi	*Pinctada fucata*, *C. gigas*, *M. galloprovincialis*, *Venerupis philippinarum*, *Suculus diversicolor:* Adults: Mortalities, reduced filtration rate. Larvae: activity rate, development rate and survival reduced	Field blooms, bloom water, whole cell culture	[29,85,178,179,180]
		*H. illdefina* (Herman & Sweeney) Morrill & Loeblich III	None known		[114,181]
	*Karlodinium*	*K. veneficum* (Ballentine) Larsen	Fin fish mortalities	Field bloom, whole cell culture, cell lysate	[182,183,184]
			*C. virginica* & *C. ariakensis* larvae, spat & juveniles: increased mortalities & abnormalities, reduction in swimming and activity, reduced growth rates.	Whole cell culture	[185,186,187,188,189]
			Juvenile & adult *Mytilus edulis* & *Mercenaria mercenaria*: increased hemocyte phagocytosis and ROS production, reduced growth rates	Whole cell culture	[188,190]
	*Pfiesteria*	*Pfiesteria* spp.	Fin fish mortalities	Field blooms	[191,192]
		*P. piscicida* Steidinger & Burkholder	*A. irradians*, *C. gigas* & *C. virginica* larvae: mortalities	Whole cells	[193,194]
		*P. shumwayae* Glasgow & Burkholder (syn. *Pseudopfiesteria shumwayae* (Glasgow & Burkholder))	Ichthyotoxic in vitro	Whole cell culture	[195]
			*A. irradians*, *C. virginica*, *M. mercenaria*, *Perna viridis:* mortalities of larvae & adults	Whole cell culture	[196]
	*Prorocentrum*	*P. rathymum* Loeblich, Shirley & Schmidt	*C. gigas* spat: mortality	Methanol extracts	[197]
**Haptophyceae**	*Chrysochromulina*	*C. leadbeateri* Estep, Davis, Hargreaves & Sieburth	Mortalities of *Salmo salar*	Field bloom	[27,198]
	*Pavlomulina*	*P. ranunculiformis* Sym, Pienaar & Kawachi	Attaching to *C. gigas* larvae	whole cell culture	[28]
**Raphidophyceae**	*Chattonella*	*C. antiqua* (Hada) Ono (syn. *C. marina* var. *antiqua* (Hada) Demura & Kawachi)	Fin fish mortalities	Field bloom	[94,199]
			Mortalities *Thunnus maccoyii*	Field bloom	[200,201]
			*Pinctada fucata martensii*: reduced sperm swimming velocity, increased larval mortalities & abnormalities, reduced activity	Whole cell culture	[124,125]

#: Species classifications based on AlgaeBase (Guiry in [130]). *Alexandrium* classification as for [131].

## Data Availability

Not applicable.

## Supplementary Materials

The following supporting information can be downloaded at: https://www.mdpi.com/article/10.3390/toxins14050341/s1, Table S1: Details of the primers used in this study for the high-throughput sequencing metabarcoding analyses [246,247,248]; Table S2: Sea water samples from around the South Island of New Zealand, collected as part of the New Zealand Marine Phytoplankton Monitoring Programme and containing cells of potentially ichthyotoxic species were analysed using high-throughput sequencing metabarcoding using the four primer pairs listed in Supplementary Table S1. The resulting taxonomic classifications of eukaryotic phytoplankton types that are associated with harmful effects are listed. No raphidophytes or dictyochophytes were detected.
