# Supplementary material for: Effects of Harmful Algal Blooms on Fish and Shellfish Species: A Case Study of New Zealand in a Changing Environment
Source: Toxins (Basel). 2022 May 14;14(5):341. doi: 10.3390/toxins14050341 (PMC9147682; doi:10.3390/toxins14050341)
Supplement: Supplementary file 1 [file toxins-14-00341-s001.zip › toxins-1705024-supplementary.pdf]

Supplementary Material

Table S1. Details of the primers used in this study for the high-throughput sequencing metabarcoding analyses.

Primer name	Primer sequence (5′-3′)	Target gene region	Reference
Uni18SF	AGG GCA AKY CTG GTG CCA GC	V4 region of the eukaryote nuclear 18S ribosomal RNA (18S)	246
Uni18SR	GRC GGT ATC TRA TCG YCT T		
Hap454-F	GCGGTAATTCCAGCTCCAA	Haptophyta V4 region of the 18S ribosomal RNA (18S haptophytes)	247
Hap454-R	GATCAGTGAAAACATCCCTGG		
D1R-F	ACCCGCTGAATTTAAGCATA	Dinophyceae D1-D2 region of the 28S ribosomal RNA (28S)	248
305-R	TTTAAYTCTCTTTYCAAAGTCC		241
Lhapto8-F	CCATCTCATCCCTGCGTGTCTCCGAC	Haptophyta D1-D2 region of the 28S ribosomal RNA (28S haptophytes)	242
Lhapto20R_bis-R	TCAGACTCCTTGGTCCGTGTTTCT		

Table S2. Sea water samples from around the South Island of New Zealand, collected as part of the New Zealand Marine Phytoplankton Monitoring Programme and containing cells of potentially ichthyotoxic species were analysed using high-throughput sequencing metabarcoding using the four primer pairs listed in Supplementary table 1. The resulting taxonomic classifications of eukaryotic phytoplankton types that are associated with harmful effects are listed. No raphidophytes or dictyochophytes were detected.

**Universal 18S ribosomal RNA - Diatoms**	**Universal 18S ribosomal RNA - Dinoflagellates**	**Specific 18S ribosomal RNA - Haptophytes**	**Specific 28S ribosomal RNA – Dinoflagellates**	**Specific 28S ribosomal RNA - Haptophytes**
Actinocyclus curvatulus	*Akashiwo sanguinea*	*Algirosphaera robusta*	*Akashiwo sanguinea*	*Chrysochromulina spinifera*
Cerataulina pelagica	*Alexandrium affine*	*Braarudosphaera bigelowii*	*Alexandrium affine*	*Chrysochromulina camella*
Chaetoceros spp.	*Alexandrium* spp.	*Calcihaptophycidae sp.*	*Alexandrium pacificum*	*Chrysochromulina cymbium*
Chaetoceros convolutus	*Alexandrium margalefii*	*Chrysochromulina spinifera*	*Alexandrium margalefii*	*Chrysochromulina simplex*
Chaetoceros danicus	*Alexandrium minutum*	*Chrysochromulina acantha*	*Alexandrium minutum*	*Chrysochromulina* spp.
Chaetoceros diadema	*Alexandrium ostenfeldii*	*Chrysochromulina rotalis*	*Alexandrium ostenfeldii*	*Helladosphaera* sp.
Chaetoceros elegans	*Alexandrium pacificum*	*Chrysochromulina scutellum*	*Alexandrium* spp.	*Imantonia rotunda*
Chaetoceros muellerii	*Amoebophyra* spp.	*Chrysochromulina simplex*	*Amphidinium crissum*	*Phaeocystis* sp.
Chaetoceros rostratus	*Amphidoma languida*	*Chrysochromulina spp.*	*Amphidoma languida*	*Prymnesium* spp.
Chaetoceros rotosporus	*Amphisoleniaceae* sp.	*Coccolithus braarudii*	*Amylax triacantha*	
Chaetoceros tenuissimus	*Amylax buxus*	*Diacronema ennorea*	*Archaeperidinium constrictum*	
Chrysophyceae spp.	*Amylax triacantha*	*Dicrateria rotunda*	*Azadinium concinnum*	
Chrysophyta sp.	*Ansanella granifera*	*Dicrateria sp.*	*Azadinium cuneatum*	
Coscinodiscus wailesii	*Apocalathium aciculiferum*	*Exanthemachrysis sp.*	*Azadinium poporum*	
Ectocarpus siliculosus	*Archaeperidinium minutum*	*Gephyrocapsa muellerae*	*Azadinium spinosum*	
Guinardia delicatula	*Asulcocephalium miricentonis*	*Gephyrocapsa oceanica*	*Azadinium trinitatum*	
Leptocylindrus convexus	*Azadinium dexteroporum*	*Haptolina* spp.	*Barrufeta bravensis*	
Leptocylindrus sp.	*Azadinium trinitatum*	*Haptophyta* spp.	*Biecheleria cincta*	
Minutocellus polymorphus	*Biecheleria cincta*	*Helicosphaera carteri*	*Biecheleriopsis adriatica*	

Paraphysomonas butcheri	*Biecheleriopsis adriatica*	*Helladosphaera* sp.	*Boreadinium breve*	
Paraphysomonas foraminifera	*Blastodinium mangini*	*Phaeocystis globosa*	*Cachonina hallii*	
Paraphysomonas imperforata	*Ceratoperidinium falcatum*	*Phaeocystis* spp.	*Calciodinellum albatrosianum*	
Paraphysomonas sp.	*Chytriodinium roseum*	*Prymnesiaceae* sp.	*Ceratium fusus*	
Parmales sp.	*Crypthecodinium sp.*	*Prymnesiophyceae* spp.	*Ceratium tripos*	
Pelagomonas calceolata	Dino-Group-I sp.	*Prymnesium* sp.	*Ceratoperidinium falcatum*	
Phaeophyceae sp.	Dino-Group-II sp.	*Scyphosphaera apsteinii*	*Ceratoperidinium* sp.	
Pseudo-nitzschia pseudodelicatissima	Dino-Group-III sp.	*Syracosphaerales* sp.	*Dinophysis* spp.	
Thalassiosira tenera	*Dinophysis* spp.		*Dissodinium pseudolunula*	
	Diplopsalis caspica		*Dissodinium* sp.	
	Dissodinium pseudolunula		*Ensiculifera aff. imariensis*	
	Euduboscquella crenulata		*Ensiculifera* cf. *loeblichii*	
	Fragilidium sp.		*Fragilidium duplocampanaeforme*	
	Fragilidium subglobosum		*Glenodinium inaequale*	
	Gonyaulax fragilis		*Gonyaulax baltica*	
	Gonyaulax polygramma		*Gonyaulax* cf. *spinifera*	
	Gonyaulax sp.		*Gonyaulax digitale*	
	Gonyaulax spinifera		*Gonyaulax elongata*	
	Gotoius excentricus		*Gymnodinium agaricoides*	
	Gymnodinium aureolum		*Gymnodinium aureolum*	
	Gymnodinium dorsalisulcum		*Gymnodinium* spp.	
	Gymnodinium microreticulatum		*Gymnodinium dorsalisulcum*	
	Gymnodinium smaydae		*Gymnodinium fuscum*	
	Gymnodinium sp.		*Gymnodinium litoralis*	

	Gyrodinium dominans		*Gymnodinium microreticulatum*	
	Gyrodinium fusiforme		*Gymnodinium* spp.	
	Gyrodinium gutrula		*Gyrodinium fusiforme*	
	Gyrodinium helveticum		*Gyrodinium moestrupii*	
	Gyrodinium heterogrammum		*Gyrodinium rubrum*	
	Gyrodinium moestrupii		*Gyrodinium spirale*	
	Gyrodinium sp.		*Heterocapsa orientalis*	
	Gyrodinium spirale		*Heterocapsa rotundata*	
	Haplozoon praxillellae		*Heterocapsa* spp.	
	Heterocapsa nei/rotundata		*Islandinium minutum*	
	Heterocapsa pygmaea		*Kapelodinium vestifici*	
	Heterocapsa sp.		*Karenia mikimotoi*	
	Heterocapsa triquetra		*Karenia umbella*	
	Islandinium tricingulatum		*Karlodinium* sp.	
	Karenia mikimotoi		*Karlodinium armiger*	
	Karlodinium veneficum		*Karlodinium ballantinum*	
	Kofoidinium pavillardii		*Karlodinium decipiens*	
	Lepidodinium chlorophorum		*Karlodinium gentienii*	
	Luciella sp.		*Karlodinium veneficum*	
	Noctiluca scintillans		*Katodinium glaucum*	
	Paragymnodinium shiwhaense		*Lebouraia pusilla*	
	Paulsenella vonstoschii		*Lepidodinium chlorophorum*	
	Pelagodinium sp.		*Lepidodinium viride*	
	Pentapharsodinium sp.		*Oblea rotunda*	
	Pentapharsodinium tyrrhenicum		*Oxyphysis oxytoxoides*	

	Pfiesteria sp.		*Peridiniopsis brodyi*	
	Pheopolykrikos beauchampii		*Peridinium aciculiferum*	
	Polykrikos kofoidii		*Polarella glacialis*	
	Prorocentrum spp.		*Polykrikos kofoidii*	
	Proterythropsis sp.		*Prorocentrum spp.*	
	Protoceratium reticulatum		*Prorocentrum triestinum*	
	Protodinium simplex		*Protodinium* sp.	
	Protoperidinium americanum		*Protoperidinium americanum*	
	Protoperidinium bipes		*Protoperidinium biconicum*	
	Protoperidinium claudicans		*Protoperidinium excentricum*	
	Protoperidinium conicum		*Protoperidinium lewisiae*	
	Protoperidinium denticulatum		*Protoperidinium parthenopes*	
	Protoperidinium fusiforme		*Protoperidinium punctulatum*	
	Protoperidinium monovelum		*Protoperidinium steidingerae*	
	Protoperidinium pellucidum		*Scrippsiella donghaienis*	
	Protoperidinium punctulatum		*Scrippsiella lachrymosa*	
	Protoperidinium sp.		*Scrippsiella ramonii*	
	Qia lebouriae		*Scrippsiella trochoidea*	
	Scrippsiella acuminata		*Symbiodinium* sp. type A	
	Scrippsiella precaria		*Symbiodinium voratum*	
	Spatulodinium pseudonoctiluca		*Takayama acrotrocha*	
	Stoeckeria algicida		*Thoracosphaera* sp.	

	Symbiodinium sp. Clade A		*Torodinium robustum*	
	Syndinium sp.		*Tyrannodinium berolinense*	
	Thecadinium kofoidii		*Vulcanodinium rugosum*	
	Torodinium robustum		*Warnowia* sp.	
	Tovellia aveirensis		*Woloszynskia halophila*	
	Tripos concilians			
	Tripos furca			
	Tripos fusus			
	Warnowia sp.			
	Woloszynskia halophila			
	Woloszynskia sp.			

246 Zhan, A.; Hulák, M.; Sylvester, F.; Huang, X.; Adebayo, A.A.; Abbott, C.L.; Adamowicz, S.J.; Heath, D.D.; Cristescu, M.E.; MacIsaac, H.J.; et al. High sensitivity of 454 pyrosequencing for detection of rare species in aquatic communities. *Methods Ecol. Evol.* **2013**, *4*, 558–565.

247 Egge, E.; Bittner, L.; Andersen, T.; Audic, S.; de Vargas, C.; Edvardsen, B. 454 pyrosequencing to describe microbial eukaryotic community composition, diversity and relative abundance: A test for marine haptophytes. *PLoS ONE* **2013**, *8*, e74371.

248 Scholin, C.A.; Herzog, M.; Sogin, M.; Anderson, D.M. Identification of group-specific and strain-specific genetic-markers for globally distributed *Alexandrium* (Dinophyceae). II. Sequence analysis of a fragment of the LSU rRNA gene. *J. Phycol*. **1994**, *30*, 999–1011.
